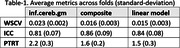# Choosing wisely: Evaluating reference regions for improved longitudinal 18F‐AV‐1451 tau PET consistency

**DOI:** 10.1002/alz.093967

**Published:** 2025-01-09

**Authors:** Isaac Llorente Saguer, Neil P Oxtoby

**Affiliations:** ^1^ UCL Centre for Medical Image Computing, Department of Computer Science, University College London, London United Kingdom; ^2^ University College London, London United Kingdom

## Abstract

**Background:**

Positron emission tomography (PET) can facilitate precise spatial and temporal measurement of Alzheimer’s disease pathology in the brain. The default quantification measure is the standardized uptake value ratio (SUVR) of radiotracer uptake in a target region of interest normalised by uptake in a reference region. Evaluations of new radiotracers commonly assess test‐retest consistency in scans with no pathological changes, but rarely question the SUVR paradigm. Notably, even the Centiloid/CenTauR scales depend on SUVR. This study aims to quantitatively assess the impact of different reference regions on 18F‐AV‐1451 tau‐PET scans.

**Method:**

We analysed SUVR data from 399 participants in the Alzheimer’s Disease Neuroimaging Initiative (ADNI) having at least two 18F‐AV‐1451 tau‐PET scans (199 CU, 149 MCI, 51 AD). Across multiple target regions of interest and common reference regions we compared SUVR longitudinal consistency for this radiotracer using median percent test‐retest (PTRT), average intraclass correlation coefficient (ICC), and average within‐subject coefficient of variation (WSCV). Target regions considered: cerebellum, brainstem, eroded subcortical white‐matter. Reference regions considered: inferior cerebellum grey‐matter, composite (cerebellum, eroded subcortical white‐matter, brainstem), and a data‐driven linear combination of these regions. Four‐fold cross‐validation stratified by diagnostic group was used to train the linear model (optimizing the three metrics factored), using 75% of data for training and 25% for testing.

**Result:**

Table‐1 presents test‐retest performance across reference regions. The inferior cerebellum gray‐matter was the worst‐performing reference region with only 2.2% test‐retest consistency. The composite reference region produced 26% lower (better) PTRT, 33% lower (better) WSCV and 5% higher (better) ICC. Our data‐driven linear combination of reference regions outperformed inferior cerebellum gray‐matter by 32% PTRT, 37% WSCV and 3% ICC.

**Conclusion:**

Longitudinal consistency varied based on the reference region chosen. The composite reference region and the linear model both outperformed the inferior cerebellum grey‐matter. This study emphasizes the importance of selecting an appropriate reference region for accurate assessment and tracking of Alzheimer’s disease pathology in 18F‐AV‐1451 tau PET scans, in line with other studies (Young et al. 2021).